# A Case of Delayed-Onset Stent-Induced Dissection of the Internal Carotid Artery After Stenting for Near-Occlusion

**DOI:** 10.7759/cureus.83855

**Published:** 2025-05-10

**Authors:** Eisaku Terada, Aya Ozaki, Kazuhiro Tohara, Takamitsu Iwata, Ryuichiro Kajikawa, Takashi Tsuzuki

**Affiliations:** 1 Department of Neurosurgery, Sakai City Medical Center, Sakai, JPN

**Keywords:** carotid artery stenting (cas), iatrogenic dissection, internal carotid artery (ica), near occlusion, vista

## Abstract

Internal carotid artery near-occlusion (ICANO) is characterized by severe internal carotid artery (ICA) stenosis with distal collapse. Although carotid artery stenting (CAS) is a treatment option, its efficacy and the associated complications remain controversial. Herein, we report a case of ICANO treated with CAS complicated by delayed occlusive dissection at the distal stent edge, which was successfully managed with additional stenting.

A 52-year-old man with hypertension, depression, and heavy smoking was referred for low left ICA signal intensity on magnetic resonance angiography (MRA). Carotid ultrasound and computed tomography angiography (CTA) confirmed severe stenosis with distal vessel collapse. Digital subtraction angiography (DSA) showed a delayed flow and collateral circulation through the anterior and posterior communicating arteries. The patient was diagnosed with asymptomatic ICANO and underwent CAS to prevent stroke. An open-cell stent was then deployed. While postprocedural imaging confirmed ICA patency, full vessel expansion was not achieved, leading to a diameter mismatch at the distal stent edge. The patient initially remained asymptomatic; however, mild posterior neck pain occurred on postoperative day 4. On day 5, carotid ultrasonography revealed an ICA occlusion. Urgent CTA and MRA revealed poor ICA visualization, and three-dimensional T1-weighted imaging (3D T1-volume isotropic turbo spin-echo acquisition (VISTA)) revealed a dissection flap extending to the proximal petrous ICA, causing severe stenosis. Urgent DSA confirmed a severely delayed contrast flow immediately distal to the stent. Endovascular treatment with closed-cell stents successfully restored ICA patency. The patient was discharged without neurological deficits or neck pain, and follow-up imaging confirmed sustained vessel patency.

This case highlights that delayed arterial dissection is a rare but serious complication of CAS in patients with ICANO and vessel collapse. Stent selection and vessel diameter mismatch may contribute to intimal injury, which requires careful procedural planning.

## Introduction

Internal carotid artery near-occlusion (ICANO) is characterized by severe stenotic lesions in the internal carotid artery (ICA). Imaging findings typically include delayed contrast filling, collateral circulation through the posterior communicating artery (Pcom) and anterior communicating artery (Acom), and collapse of the distal internal carotid artery [1]. Treatment options include best medical therapy (BMT), carotid endarterectomy (CEA), and carotid artery stenting (CAS); however, no consensus has been reached on the optimal management approach [2].

There are two types of ICA dissection: spontaneous and iatrogenic. Iatrogenic arterial dissection is a recognized complication of neuroendovascular therapy, particularly in procedures involving mechanical thrombectomy and balloon-guiding catheters [3,4]. ICA dissection is typically caused by a tear in the intima and media layers of the artery, leading to bleeding within the vessel wall and resulting in luminal compromise [5]. However, reports of arterial dissection caused directly by a stent during CAS, where the stent is in contact with the intima of the artery, are rare.

Here, we report a rare case of ICANO treated with CAS, in which delayed occlusive dissection occurred in the distal portion of the stented artery. Despite this complication, successful recanalization was achieved through additional stent placement.

## Case presentation

**Figure 1:**
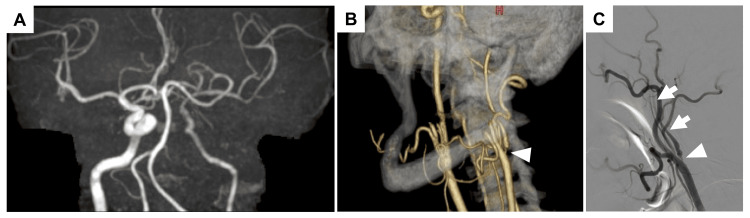
Imaging findings of near occlusion in the left ICA A: Head MRA showing poor visualization of the left ICA; B: Neck CTA demonstrating near occlusion of the left ICA. The arrowheads indicate the point of maximal stenosis; C: Left CCA angiogram showing near-occlusion and collapse of the left ICA. The arrowhead marks the point of maximal stenosis, and the arrows indicate a collapsed segment of the left ICA. ICA: internal carotid artery; CCA: common carotid artery; CTA: computed tomography angiography; MRA: magnetic resonance angiography

A 52-year-old man with a history of hypertension, depression, and heavy smoking was referred to our hospital after a brain magnetic resonance imaging (MRI) revealed reduced blood flow in the left ICA. Although MRI of the head showed no infarction in the left hemisphere, magnetic resonance angiography (MRA) revealed decreased signal intensity in the left ICA (Figure 1A). Carotid ultrasound revealed stenosis at the origin of the left ICA, with a peak systolic velocity of 167 cm/s. Computed tomography angiography (CTA) confirmed severe stenosis with distal vessel collapse (Figure 1B). Digital subtraction angiography (DSA) revealed severe stenosis of the left ICA with slow flow and distal collapse (Figure 1C). Collateral circulation through the anterior and posterior communicating arteries supplies the left cerebral hemisphere. Black blood MRA suggested a stable plaque, and single-photon emission computed tomography (SPECT) showed only mild cerebral blood flow reduction. The transthoracic echocardiography revealed no abnormalities. The patient was diagnosed with an asymptomatic ICANO. Aspirin and clopidogrel were administered, and CAS was planned for stroke prevention.

**Figure 2:**
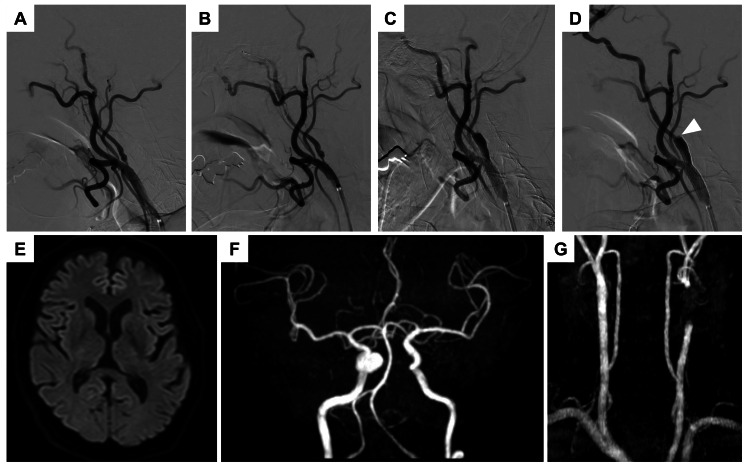
Intraoperative angiograms and postoperative imaging findings of CAS A: Preoperative left CCA angiogram; B: Left CCA angiogram showing the protection filter placed in the left ICA; C: Left CCA angiogram immediately after placement of an open-cell stent; D: Final angiogram of CAS. The arrowhead indicates contrast stagnation; E: DWI performed on the day after CAS, showing no high-intensity lesions in the left cerebral hemisphere; F-G: Head and neck MRA performed the day after CAS, demonstrating normalization of the ICA visualization. CAS: carotid artery stenting; CCA: common carotid artery; ICA: internal carotid artery; DWI: diffusion-weighted imaging; MRA: magnetic resonance angiography

Under local anesthesia, an 8F Optimo guiding catheter (Tokai Medical Products, Aichi, Japan) was placed in the left common carotid artery (CCA) via the right transfemoral approach (Figure 2A). A FilterWire EZ (Boston Scientific, Marlborough, Massachusetts, USA) was deployed in the cervical ICA for embolic protection (Figure 2B). Balloon angioplasty was performed with a Coyote 3.0m × 40 mm balloon (Boston Scientific) at 6 atm for 30 s. A 9.0 mm × 40 mm open-cell Precise stent (Cordis, Miami, Florida, USA) was deployed (Figure 2C), followed by post-dilation using a 4.0 20 mm Sterling balloon (Boston Scientific) at 6 atm for 30 seconds. The final angiogram and intravascular ultrasound confirmed the absence of thrombus or plaque protrusion within the stented lumen. While the distal ICA collapse was relieved, full expansion was not achieved, resulting in a significant diameter mismatch at the distal stent edge with mild contrast stagnation (Figure 2D). No postoperative neurological deficits or neck pain were observed.

Follow-up MRI on postoperative day 1 revealed no evidence of infarction or ICA dissection, and MRA confirmed good ICA patency (Figures 2E-2G). SPECT on postoperative day 2 showed no hyperperfusion or reduction in cerebral blood flow.

**Figure 3:**
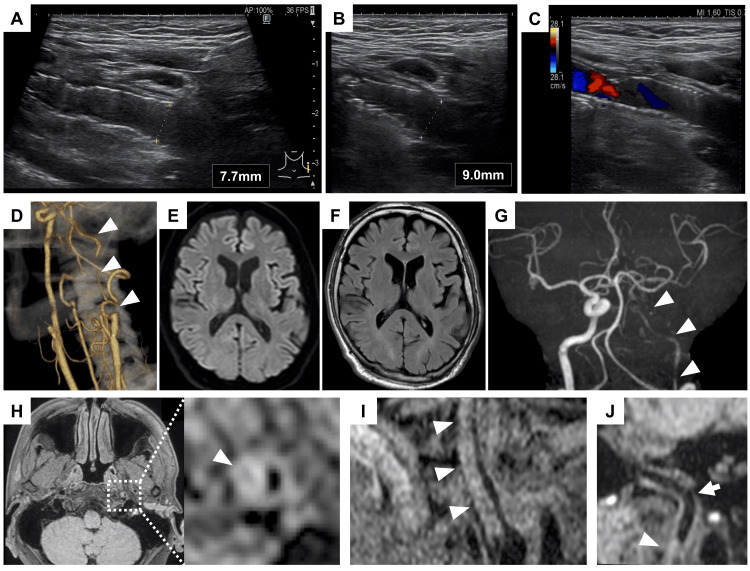
Imaging findings left ICA dissection A-B: Carotid ultrasound on postoperative day 5 showing dilation of the distal stent segment and the ICA beyond the stent; C: Color Doppler ultrasound of the neck on postoperative day 5 showing absence of blood flow in the ICA distal to the stent; D: Neck CTA on postoperative day 5 shows poor visualization of the ICA distal to the stent. Arrowheads indicate the ICA with reduced visibility; E-F: DWI and FLAIR on postoperative day 5, showing no high-intensity lesions in the left cerebral hemisphere; G: Head MRA showing poor visualization of the left ICA; H-J: 3D-T1-VISTA reveals a crescent-shaped deformation of the arterial lumen due to the dissection flap extending from the distal stent region to the proximal petrous portion of the left ICA. Arrowheads indicate the dissection flap, and the arrow indicates the petrous portion of the left ICA. CTA: computed tomography angiography; DWI: diffusion-weighted imaging; ICA: internal carotid artery; FLAIR: fluid-attenuated inversion recovery; MRA, magnetic resonance angiography, 3D-T1-VISTA: Three-dimensional T1-weighted volume isotropic turbo spin-echo acquisition

On the morning of postoperative day 4, the patient reported mild left posterior neck pain, which was managed with analgesics. On postoperative day 5, carotid ultrasonography suggested dilatation of the distal portion of the stent and the distal ICA, accompanied by findings indicative of ICA occlusion (Figure 3A-3C), prompting urgent CTA. CTA revealed poor contrast filling beyond the distal edge of the stent (Figure 3D). Despite the persistent neck pain, no neurological deficits were observed. Head MRI revealed no acute infarction (Figures 3E, 3F); however, MRA demonstrated poor visualization of the left ICA (Figure 3G). Three-dimensional T1-weighted volume isotropic turbo spin-echo acquisition (3D-T1-volume isotropic turbo spin-echo acquisition (VISTA)) imaging showed crescentic deformation of the ICA lumen due to a dissection flap extending just beyond the distal stent edge to the proximal petrous portion, which caused severe stenosis (Figures 3H-3J). Based on these findings, occlusion or severe stenosis due to the dissection was suspected, prompting urgent DSA.

**Figure 4:**
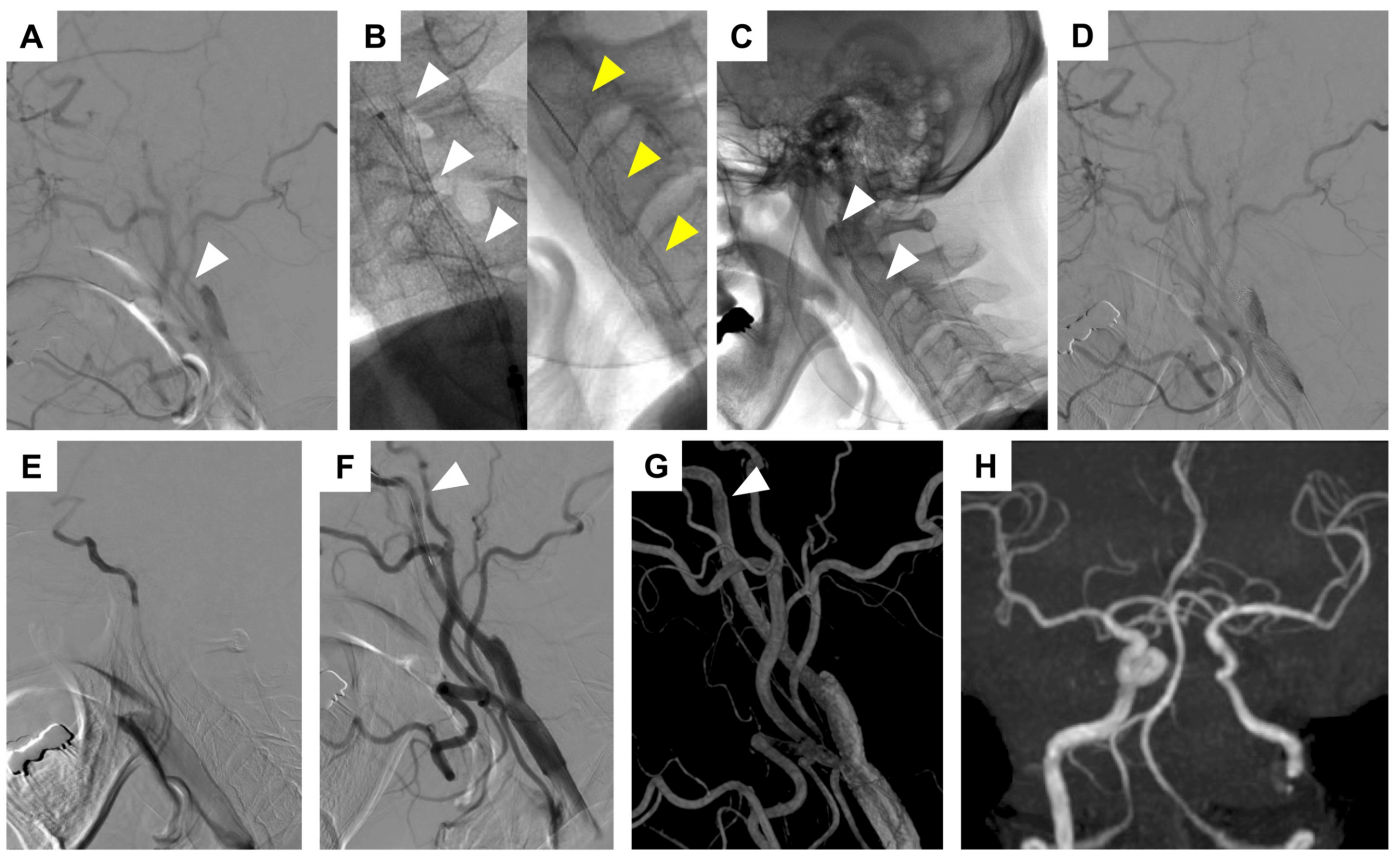
Intraoperative angiograms and postoperative imaging findings of second CAS A: Preoperative left CCA angiogram. The arrowhead indicates severely slow flow in the left ICA; B: Radiographic images immediately after stent placement and prior to the second CAS. The white arrowhead indicates the initial stent, and the yellow arrowhead indicates the stent prior to the second CAS; C: Radiographic image after stent placement. The arrowheads indicate additional closed-cell stents; D: CCA angiogram after the placement of the additional stent; E: Microcatheter angiography in the normal left ICA distal to the dissection site; F-G: Final angiogram and 3D-RA image. The arrowhead indicates the most distal part of the second closed-cell stent; H: Head MRA performed the day after the second CAS, demonstrating normalization of ICA visualization. CAS: carotid artery stenting; CCA: common carotid artery; ICA: internal carotid artery; MRA: magnetic resonance angiography; 3D-RA: three-dimensional rotational angiography

Under local anesthesia, left transfemoral DSA confirmed severely slowed contrast flow immediately distal to the stent with retrograde visualization of the distal ICA via the left ophthalmic artery (Figure 4A). Radiographic imaging demonstrated dilatation at the distal portion of the stent (Figure 4B). Given the absence of neurological deficits or infarction, endovascular revascularization was performed to restore antegrade blood flow. An 8F Optimo catheter was introduced into the left CCA. Using the true lumen as a guide, a CHIKAI 14 microwire (Asahi Intec, Nagoya, Japan) and an Excelsior SL-10 microcatheter (Stryker, Fremont, California, USA) were advanced distally, achieving access to the normal vessel. A 6.0 mm × 22 mm closed-cell Wallstent (Boston Scientific) was deployed and connected to the previously implanted stent (Figure 4C). However, contrast injection failed to visualize flow beyond the open-cell stent (Figure 4D). To assess the distal segment, the microcatheter was advanced beyond the dissection site to reconfirm normal vasculature (Figure 4E). To reinforce the distal segment, an 8.0 mm × 29 mm closed-cell Wallstent was deployed from the proximal petrous portion, successfully restoring ICA patency (Figures 4F, 4G).

Follow-up MRI on postoperative day 1 showed no new infarctions, and MRA confirmed sustained ICA patency (Figure 4H). The patient was discharged without neurological deficits or neck pain on postoperative day 11. At the one-month follow-up, carotid ultrasonography and MRA confirmed good ICA patency.

## Discussion

Arterial dissection is a rare complication of CAS. Previous reports have described dissections caused by protection balloon inflation or delayed dissections after stent placement in tortuous ICA segments [6,7]. However, to the best of our knowledge, this is the first report of delayed distal dissection following CAS in a patient with ICANO and vessel collapse.

The degree of ICA stenosis correlates with the risk of ipsilateral ischemic stroke [8]. However, the risk of stroke in patients with near occlusion remains controversial. Current guidelines recommend BMT for occlusion and near-occlusion [9]; however, these recommendations are based on older data. Recent studies suggest that CEA or CAS may be more effective than BMT in reducing stroke/death rates [10], with some studies reporting similar outcomes between ICANO and conventional stenosis [11]. Advances in interventional techniques and devices may further clarify the benefits of surgical intervention; however, this report shows the risk associated with performing a preventive procedure on an asymptomatic individual. This risk should be carefully considered when expanding the indications for such procedures.

Open-cell stents exert a strong radial force [12]. In this case, given the severe stenosis in a relatively young patient, an open-cell stent was used to maximize vessel expansion. Even in collapsed lesions, the ICA diameter can recover after stent placement [13]. Intraoperatively, the significant difference in vessel diameter between the stented segment and the distal ICA was not considered problematic. However, retrospectively, there was slight contrast stagnation at the distal portion of the stent, suggesting possible intimal injury at that time. Additionally, as self-expanding stents continue to expand over the course of three months post-procedure [14], progressive intimal injury due to mechanical expansion may have contributed to delayed dissection. Head and neck MRA performed on the day after the initial CAS revealed normal visualization of the distal left ICA, suggesting that the dissection had not yet progressed. The patient's posterior neck pain, which began on postoperative day 4, likely marked the onset of dissection. The following day, the carotid ultrasound revealed significant stenosis at the distal end of the stent.

The primary issue, in this case, was that placing a high-expansion open-cell stent in a collapsed ICANO resulted in a substantial mismatch in the vessel diameter, leading to intimal injury distal to the stent. Possible preventive strategies include using a closed-cell stent with a milder radial force or performing a staged angioplasty to achieve gradual dilation of the severe stenosis [15]. Additionally, choosing CEA, which has been reported to have outcomes comparable to those of CAS, could also be considered [16].

Medical management with antiplatelet or anticoagulation therapy is commonly employed [17]. However, in cases of severe stenosis or refractory medical treatment, endovascular treatment is often performed using stents such as Wallstent (Boston Scientific) or Wingspan (Boston Scientific) [18-20]. Given the severe stenosis and the absence of ischemic stroke, early intervention was deemed appropriate in this case.

A 3D T1-VISTA is reportedly useful in the diagnosis of ICA dissection [21]. In the present case, preoperative 3D T1-VISTA imaging confirmed that the distal end of the dissected segment was proximal to the petrous portion of the ICA. During the procedure, a microcatheter contrast injection at the distal ICA confirmed true lumen access, and preoperative MRI findings allowed for precise localization of the distal end of the dissection. These factors facilitated the decision to place a second stent and accurately determined its placement, ultimately leading to successful recanalization.

## Conclusions

We report a rare case of ICANO treated with CAS, complicated by delayed obstructive dissection distal to the stent. The use of an open-cell stent likely contributed to a mismatch in vessel diameter at the distal edge, resulting in intimal injury and subsequent dissection. Postoperative neck pain was an early clinical sign, and timely imaging and endovascular reintervention successfully achieved recanalization without neurological deficits.

In CAS for ICANO, careful consideration of distal vessel morphology is essential when selecting a stent. Open-cell stents may be associated with distal injury, particularly in collapsed or narrowed segments. Therefore, alternative strategies, such as using closed-cell stents or performing staged angioplasty, should be considered. This case demonstrates the value of high-resolution vessel wall imaging, such as 3D-T1-VISTA, in the accurate diagnosis and planning of treatment strategies.
